# Steady state free precession cardiovascular magnetic resonance: accuracy of left and right ventricular functional assessment in children

**DOI:** 10.1186/1532-429X-15-S1-T9

**Published:** 2013-01-30

**Authors:** Jennifer A Bryant, Keith Godfrey, Mark A Hanson, Lucy Davies, Alison Fletcher, Charles Peebles

**Affiliations:** 1 NIHR Southampton Biomedical Research Centre, University of Southampton and University Hospital Southampton NHS Foundation Trust, Southampton, UK; 2 Cardiothoracic Radiology, University Hospital Southampton NHS Foundation Trust, Southampton, UK; 3 Institute of Developmental Sciences, University of Southampton, Southampton, UK; 4 MRC Lifecourse Epidemiology Unit, Southampton, UK

## Background

Cardiovascular Magnetic Resonance (CMR) is recognised as the gold standard technique for the assessment of cardiovascular function in adults. In children, image quality is dependent on compliance with motion control and breath-hold instructions. Inability to maintain repeated breath-holds in a consistent phase of respiration can result in respiratory misregistration errors. There is limited data on the accuracy of CMR in children.

## Methods

CMR was performed on 198 healthy 9 year old children as part of a study of developmental influences on cardiovascular structure and function. Contiguous steady state free precession cine images were acquired in the short axis (SA) plane. Data sets were analysed using a manual technique (Osirix) to calculate left ventricular (LV) and right ventricular (RV) stroke volumes (SV). Papillary muscles and trabeculae were excluded from the blood pool. LV and RV SVs were compared with flow data derived from phase contrast velocity flow mapping sequences through the aortic root and the main pulmonary artery respectively.

## Results

There were good correlations between aortic flow and LV SV (r=0.69, p<0.0001, n=198), and between pulmonary flow volume and RVSV (r=0.64, p<0.0001, n=189). Using the Bland Altman method the mean difference between LVSV and aortic flow was 10ml (95%CI 9 to 11ml). Mean difference between RV SVs and MPA flow was 12ml (95%CI 10.8 to 13.2ml). There was a strong correlation between aortic and pulmonary flow volumes (r= 0.87, p<0.0001, n=198). The mean difference between aortic and MPA flow volumes was 3.4ml.

## Conclusions

Children at the age of 9 years can comply with multiple breath-hold requirements, allowing accurate assessment of cardiovascular function using steady state free precession CMR. Although less accurate than in adults, useful measurements can be obtained at this age for research studies as there is a good correlation between flow derived SVs and those derived from the SA cine stack.

## Funding

This work was supported by funding from the British Heart Foundation and the National Institute for Health Research (NIHR Southampton Biomedical Research Centre).

